# Small Molecule Inhibitors of MERTK and FLT3 Induce Cell Cycle Arrest in Human CD8^+^ T Cells

**DOI:** 10.3390/vaccines9111294

**Published:** 2021-11-08

**Authors:** Richard M. Powell, Marlies J. W. Peeters, Anne Rahbech, Pia Aehnlich, Tina Seremet, Per thor Straten

**Affiliations:** 1 National Center for Cancer Immune Therapy (CCIT-DK), Department of Oncology, University Hospital Herlev, 2730 Herlev, Denmark; richardpowell1000@gmail.com (R.M.P.); marlies.peeters@regionh.dk (M.J.W.P.); anne.rahbech@regionh.dk (A.R.); pia.aehnlich@regionh.dk (P.A.); tina.juanita.seremet@regionh.dk (T.S.); 2 Inflammation and Cancer Group, Department of Immunology and Microbiology, University of Copenhagen, 2200 Copenhagen, Denmark

**Keywords:** MERTK, FLT3, CD8^+^ T cells, T lymphocytes, receptor tyrosine kinases, small molecule inhibitors

## Abstract

There is an increasing interest in the development of Receptor Tyrosine Kinases inhibitors (RTKIs) for cancer treatment, as dysregulation of RTK expression can govern oncogenesis. Among the newer generations of RTKIs, many target Mer Tyrosine Kinase (MERTK) and Fms related RTK 3 (FLT3). Next to being overexpressed in many cancers, MERTK and FLT3 have important roles in immune cell development and function. In this study, we address how the new generation and potent RTKIs of MERTK/FLT3 affect human primary CD8^+^ T cell function. Using ex vivo T cell receptor (TCR)-activated CD8^+^ T cells, we demonstrate that use of dual MERTK/FLT3 inhibitor UNC2025 restricts CD8^+^ T proliferation at the G2 phase, at least in part by modulation of mTOR signaling. Cytokine production and activation remain largely unaffected. Finally, we show that activated CD8^+^ T cells express FLT3 from day two post activation, and FLT3 inhibition with AC220 (quizartinib) or siRNA-mediated knockdown affects cell cycle kinetics. These results signify that caution is needed when using potent RTKIs in the context of antitumor immune responses.

## 1. Introduction

Dysregulation and overexpression of Receptor Tyrosine Kinases (RTKs) by neoplastic cells often control signaling pathways responsible for many oncogenic characteristics [1]. Key factors for metabolism and growth (Pi3K, AKT, mTOR), migration (RAS/MAPK), cell signaling (STAT), and cell survival and motility (PKC) are activated by RTKs, whereby constitutive expression can govern oncogenesis [2]. For several decades, there has been great interest in the development of Receptor Tyrosine Kinase Inhibitors (RTKIs), as a form of primary or complementary clinical therapy for cancer. Early generation inhibitors with broad spectrum activity, such as dasatinib, have higher toxicity and undesirable off-target activity [3,4]. Newer generations of RTKIs, such as quizartinib (AC220) or UNC2025, demonstrate high specificity and potency against their intended targets of Fms Related Receptor Tyrosine Kinase 3 (FLT3) and Mer tyrosine kinase (MERTK), respectively [5,6]. As a member of the TAM receptor family (TYRO3, AXL, MERTK), MERTK is overexpressed in many solid cancers (such as breast cancer, colorectal cancer, and melanoma) as well as in hematological malignancies such as acute myeloid leukemia (AML) [7,8,9,10]. As cancer cells also express the MERTK ligand Protein S (PROS1) and phosphatidylserine, this auto stimulatory loop leads to increased oncogenesis. Several studies have shown that use of inhibitors specific for MERTK have a significant effect on tumor cell growth in cell lines, murine models and patient primary tumor cells [11,12,13]. In addition, inhibitors of MERTK MRX-2843 and RXDX-106 have been shown to promote anti-tumor immune responses in vivo in mice [14,15]. FLT3 is another highly attractive therapeutic target, due to the fact it is overexpressed in most AML cases with a high mutational rate [16,17]. Proven effects on tumor cell cycling by FLT3 inhibitors [18,19,20] have led to many clinical trials either as a monotherapy, or as combinatorial therapy with conventional chemotherapy, with early meta-analysis suggesting a statistically significant 17% improvement in survival [21].

MERTK and FLT3 also have key roles in immune cell development and function. MERTK is primarily known for its role in silent phagocytosis of apoptotic material by macrophages and dendritic cells [22]. Engagement of MERTK cell signaling pathways results in an immunomodulatory switch in these antigen-presenting cells [23,24]. MERTK is also expressed by human T cells, where PROS1-MERTK signaling results in increased proliferation, cytokine expression, and possible memory formation [25]. Expression of FLT3, on the other hand, is largely considered to be conserved to myeloid progenitors and some lymphoid progenitors, where FLT3 signaling drives cell differentiation and development [26,27]. Next to this, the FLT3/FLT3L axis is integral to dendritic cell maturation and its expression is preserved in mature conventional and plasmacytoid dendritic cells [28,29]. It has also been suggested that T cells express FLT3 and FLT3L, but its function in T cells has not been studied yet [30,31]. Despite the important roles of MERTK and FLT3 in immune cell development, little effort has been done to fully elucidate the effect that new generation of MERTK or FLT3 inhibitors may have on a prime component the anti-tumor immune response, namely cytotoxic CD8^+^ T cells. Recent studies have shown that MERTK inhibition is effective in tumor resolution in in vivo studies, when combining MERTK inhibition with anti-PD-1 blockade in mice [32,33,34]. As PD-1 based immunotherapy is dependent on T cell-based immune responses, thorough investigations into how MERTK and other receptor tyrosine kinase inhibitors may affect an anti-tumor T cell immune responses are essential.

In this study, we address how potent RTKIs of MERTK/FLT3 affect human primary CD8^+^ T cell function. We demonstrate that dual MERTK/FLT3 inhibitor UNC2025 restricts CD8^+^ T cell cycling by modulation of mTOR signaling. We show that activated CD8^+^ T cells express FLT3 and that FLT3 inhibition with AC220 (quizartinib) or genetic knockdown affects cell cycle kinetics. These results signify that caution is needed when using potent RTKIs in the context of antitumor immune responses.

## 2. Materials and Methods

### 2.1. T Cell Isolation and Stimulation

PBMCs from healthy donor buffy coats were isolated by gradient centrifugation and used immediately or cryopreserved for later use. Cryopreserved PBMCs were thawed and rested overnight in X-VIVO 15 medium (Lonza) and 100 U/mL IL-2 (Proleukin) prior to use. CD8^+^ T cells were isolated by negative selection (EasySep, Stemcell). Isolated CD8^+^ T cells or PBMCs were activated with anti-CD3/CD28 coated Dynabeads (Gibco, 1:1 bead to cell ratio) in X-VIVO 15 medium supplemented with 50 U/mL IL-2 and 5% heat-inactivated human AB serum (Sigma-Aldrich). Small molecule inhibitors UNC2025 (Sigma-Aldrich), MRX-2843 (Medchem Express), and AC220 (quizartinib) (Medchem Express) were added to culture day 0 at 50 nM, 250 nM, 500 nM, or 750 nM. In long term cell culture assays, fresh cell culture medium with cytokines IL-2 (100 U/mL) and IL-15 (10 ng/mL, Peprotech) were replenished every 3 days, and T cells or PBMCs were split every 7 days.

### 2.2. Flow Cytometry

To track cell proliferation, cells were labelled with CellTrace Violet proliferation dye (Invitrogen). For surface staining, cells were stained with the following fluorescently labelled antibodies: anti-CD3 (clone UCHT1, BD Biosciences), anti-CD4 (SK3, Biolegend), anti-CD8 (RPA-T8, BD Biosciences) anti-CD45RO (UCHL-1, BD Biosciences), anti-CCR7 (G043H7, Biolegend), Near IR Live/Dead (Invitrogen) and anti-MERTK (124418, R&D Systems), anti-FLT3 (4G8, BD Biosciences), anti-KLRG1 (2F1, Biolegend) and anti-PD-1 (EH12.1, BD Biosciences). For intracellular cytokine staining, cells were incubated with Brefeldin A (eBioscience) for four hours, prior to extracellular staining with anti-CD3, anti-CD8, and anti-CD137. Subsequently, cells were fixed and permeabilized (Intracellular Fixation and Permeabilization Buffer Set, eBioscience) and stained with anti-IL-2 (5344.111 BD Biosciences), anti-TNF-a (MAB11, BD Biosciences), anti- Granzyme B (GB11, Fisher Scientific), anti-Perforin (BD48, Biolegend). For phosphorylation studies, cells were fixed with a two-step fixation protocol. In short, cells stimulated for four days prior to adding 400 nM UNC2025 for two hours prior to fixation. Cells were fixed with IC Fixation Buffer (eBioscience), followed by a second fixation with 90–100% methanol. Subsequently, cells were stained with the following antibodies: anti-p-mTOR (clone MMRBY, eBioscience), anti-p38-MAPK (4NIT4KK, eBioscience), anti-p-AKT (M89-61, BD Biosciences), anti-p-ERK1/2 (MILAN8R, eBioscience), anti-p-STAT5 (SRBCZX, eBioscience), anti-CD8 (RPA-T8, BD Biosciences), and anti-CD3 (UCHT1, BD Biosciences). For cell cycle analysis, cells were labelled with BrDU for two hours prior to being prepared with BrDU-APC/7AAD cell cycle analysis kit (BD Biosciences). For detection of activated-induced cell death, cell cultures were restimulated on either day 7, 14, 21, or 28 and cultured for 48 h following restimulation. Cells were then prepared and stained with Pacific Blue™ Annexin V/SYTOX™ AADvanced™ Apoptosis Kit (Invitrogen). Sample acquisition was performed using LSR II (BD Biosciences) and data were analysed using FlowJo v10.

### 2.3. Cytokine Measurements

Levels of interferon-gamma (IFN-γ) (Invitrogen, Waltham, MA, USA) and FLT3 ligand (Abcam, Cambridge, UK) were measured using enzyme-linked immunosorbent assays (ELISA) according to manufacturer’s instructions. Results were analyzed using Epoc plate reader (BioTek, Winooski, VT, USA) and Gen5 Take3 software (v1.00.4, BioTek).

### 2.4. Real-Time qPCR

Cell pellets were obtained on day three. RNA was isolated using NuceloSpin RNA kit (Macherey-Nagel, Duren, Germany). cDNA was synthesized using SuperScript VILO cDNA Synthesis Kit (Invitrogen). qPCR was performed using AriaMX (Agilent, Santa Clara, CA, USA). Amplified products were checked by dissociation curves, and expression was normalized to a housekeeping gene. Primer sequences used for FLT3 are listed in Supplementary Table S1.

### 2.5. siRNA Knockdown of FLT3

CD8^+^ T cells were stimulated for 3 days with 25 μL/mL anti-CD3/CD28 (Immunocult, Stemcell). On day 3 cells were transfected using a set of three Stealth siRNA duplexes of FLT3 (Ambion) or with a mock siRNA of scrambled sequences. The ECM830 square wave electroporation system (BTX) was used to carry out electroporation. Cells were then put into culture for 24 h before assessing FLT3 expression and cell cycle analysis. siRNA sequences used are listed in Supplementary Table S1.

### 2.6. Statistical Analysis

Graphpad Prism (v8.00) was used for all statistical analysis. Comparisons between two groups were analyzed with two-tailed paired Student *t* test and between multiple groups using one-way ANOVA or two- way ANOVA and Bonferroni multiple comparison parameters. Data are plotted with mean and SEM with statistical significance represented as *, *p* < 0.05; **, *p* < 0.01; ***, *p* < 0.001. Used statistical tests and number of biological replicates are indicated in the figure legends.

## 3. Results

### 3.1. Dual MERTK/FLT3 Inhibitor UNC2025 Inhibits CD8^+^ T Cell Proliferation

**Figure 1:**
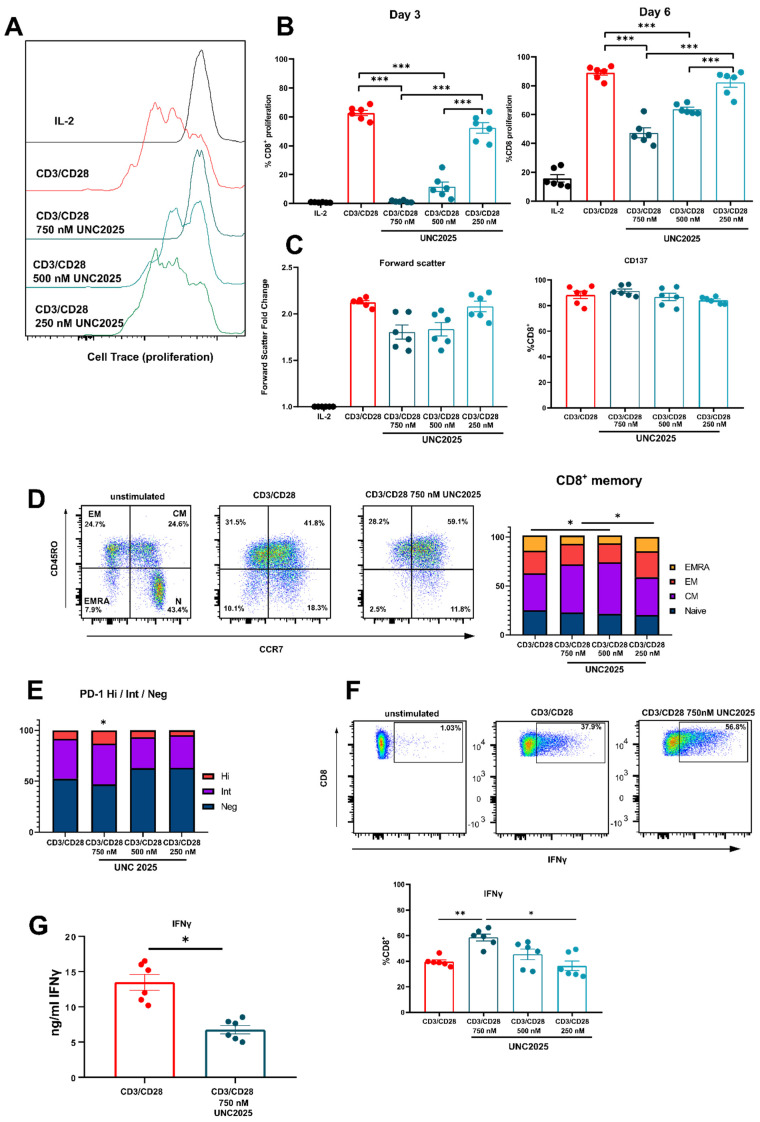
Dual MerTK/FLT3 inhibitor UNC2025 causes cell arrest of cell proliferation in CD8 T cells. Human CD8^+^ T cells were activated with CD3/CD28 + IL-2 50 U/mL and cultured in presence or absence of 750 nM, 500 nM, and 250 nM of UNC2025. (**A**) Effects of UNC2025 on CD8^+^ T cell proliferation. Representative histogram of cell division using cell trace violet as a marker of proliferation. (**B**) Percentage of proliferating CD8^+^ T cells 3 days post activation with UNC2025 added day 0; and 6 days post activation, with UNC2025 added day 3 (*n* = 6). (**C**) Fold change of MFI of forward scatter of 3 day activated CD8^+^ T cells, baseline value set using non activated CD8^+^ T cells (*n* = 6). (**D**) Comparison of percentage proportions of Naïve (CD45RO− CCR7+), Central Memory (CD45RO+ CCR7+) Effector Memory (CD45RO+ CCR7−) Terminal Effector (CD45RO− CCR7−) populations in 3-day activated CD8^+^ T cells. (**E**) Comparison of percentage proportions of PD-1 expressing populations 3-day activated CD8 T cells. (**F**) Percentage of CD137+ and IFN γ + of 3-day activated CD8^+^ T cells (*n* = 6). (**G**) IFN γ concentrations from supernatants of 3-day activated CD8^+^ T cells +/− UNC2025. Data are plotted as mean and +/− SEM where applicable. Statistical significance is calculated using Students *t* test or One-Way Anova, * *p* ≤ 0.05, ** *p* ≤ 0.01, *** *p* ≤ 0.001.

Our group recently discovered that activated human CD8^+^ T cells express MERTK, and that activation of the receptor via PROS1 initiates a costimulatory effect [25]. Therefore, we set out to investigate the effect of small molecule inhibition of MERTK on CD8^+^ T cells. Using a dual inhibitor of MERTK/FLT3 UNC2025, we cultured activated T cells in the presence of UNC2025 at concentrations 250 nM, 500 nM, and 750 nM (Figure 1A). The concentrations used reflect studies in vitro on cancer cell lines, cord blood, and bone marrow conducted by DeRyckere et al. [6]. Analysis of cell proliferation on day three of T cell activation revealed that cell division was moderately inhibited at 500 nM and completely inhibited at 750 nM UNC2025 (Figure 1B). Furthermore, when UNC2025 was added into culture following three days of activation, analysis on day six of cell culture demonstrated a significant reduction at concentration of 750 nM (approx. 50%) in cell division compared to cells cultured in the absence of the inhibitor. Arrest of cell division was not due to cell death (Supplementary Figure S1C). Cell size increase and cell activation were not affected, demonstrated by non-significant changes in fold change of forward scatter, and similar expression of CD137 respectively (Figure 1C). Assessment of memory subsets revealed small but significant increases in the central memory (CD45RO+ CCR7+) compartment with use of 500 nM and 750 nM UNC2025 (49.4% and 52.5% respectively) compared to control (37.9%) (Figure 1D). In addition, there were small but significant differences in PD-1-high expression with 750 nM UNC2025 when compared to cell culture in absence of inhibitor (13.05% and 8.16% respectively) (Figure 1E). This increased population of PD-1-high T cells is potentially linked to restriction of normal cell cycling and T cell maturation.

**Figure 2:**
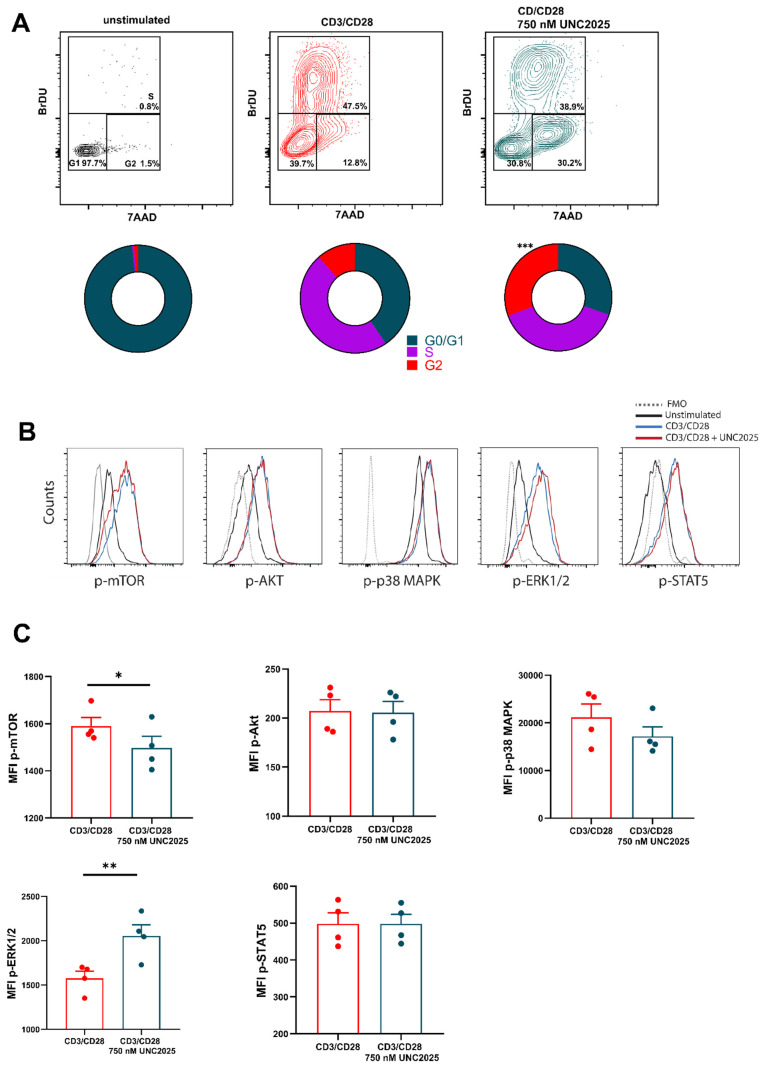
CD8^+^ T cell cycle kinetics and signaling using dual MerTK/FLT3 inhibitor **UNC2025** (**A**) Representative contour plot and comparison of percentage proportions of cell cycle populations G1 phase (7AAD low, BrDU low), S phase (7AAD med, BrDU high) G2 phase (7AAD high, BrDU high). Human CD8^+^ T cells were activated with CD3/CD28 + IL-2 50 U/mL and cultured in presence or absence of UNC2025 (*n* = 4). (**B**) Representative plot of (**C**). (**C**) MFI of phosphorylated mTOR, AKT, p38-MAPK, ERK1/2 and STAT5 in four-day stimulated T cells (with or without final two hour incubation with UNC2025) (*n* = 4). Data are plotted as mean and +/− SEM where applicable. Statistical significance is calculated using Students *t* test or One-Way Anova, * *p* ≤ 0.05, ** *p* ≤ 0.01, *** *p* ≤ 0.001.

To assess cytokine production during T cell activation in the presence of UNC2025, intracellular cytokine expression was examined. After three days of activation, use of 750 nM UNC2025 caused a significant increase in the percentage of CD8^+^ T cells expressing intracellular IFN-γ compared to control. Titration of UNC2025 to 500 nM and 250 nM resulted in the percentage of IFN-γ^+^ CD8^+^ T cells similar to that of the control, significantly lower than that seen when using UNC2025 750 nM. (Figure 1F). Other analyzed intracellular markers IL-2, TNF-α, granzyme B, and perforin were unaffected (Supplementary Figure S1E). Analysis of culture supernatants from these experiments showed a significant reduction in IFN-γ concentration in culture medium with 750 nM UNC2025 (Figure 1G). This suggests that IFN-γ production is not affected on a cell-by-cell basis following activation when UNC2025 is used in culture and is accumulated intracellularly. There is an overall decrease in the amount of IFN-γ released into the supernatant due to the smaller number of cells as cell proliferation is abrogated. Due to the potent arrest in cell division that was distinct from any impact on cell activation, we examined cell cycle kinetics of three-day activated CD8^+^ T cells using BrDU incorporation (Figure 2A). This demonstrated that, as with similar studies using UNC2025, cell cycle arrest was caused at the G2/M phase of the cell cycle, with a significant accumulation of cells in the G2 phase representing a 2.7 fold change compared to CD8^+^ T cells cultured in absence of the inhibitor. To assess pathways involved in the proliferation block upon inhibition, phosphorylation status of various signaling molecules often involved in proliferation and cell growth, was analyzed. This revealed a correlation with the mTOR pathway, as phosphorylation of mTOR was decreased, while activity of mTOR-upstream AKT remained unchanged (Figure 2B,C). Paradoxically, ERK signaling was strongly augmented, likely as a compensatory mechanism, whereas activity of p38-MAPK, and STAT5 remained unchanged (Figure 2B,C). Taken together, these data demonstrate that UNC2025-mediated inhibition of MERTK on T cells induces cell cycle arrest and is uncoupled from T cell activation.

### 3.2. FLT3 Is Expressed by Activated T Cells

**Figure 3:**
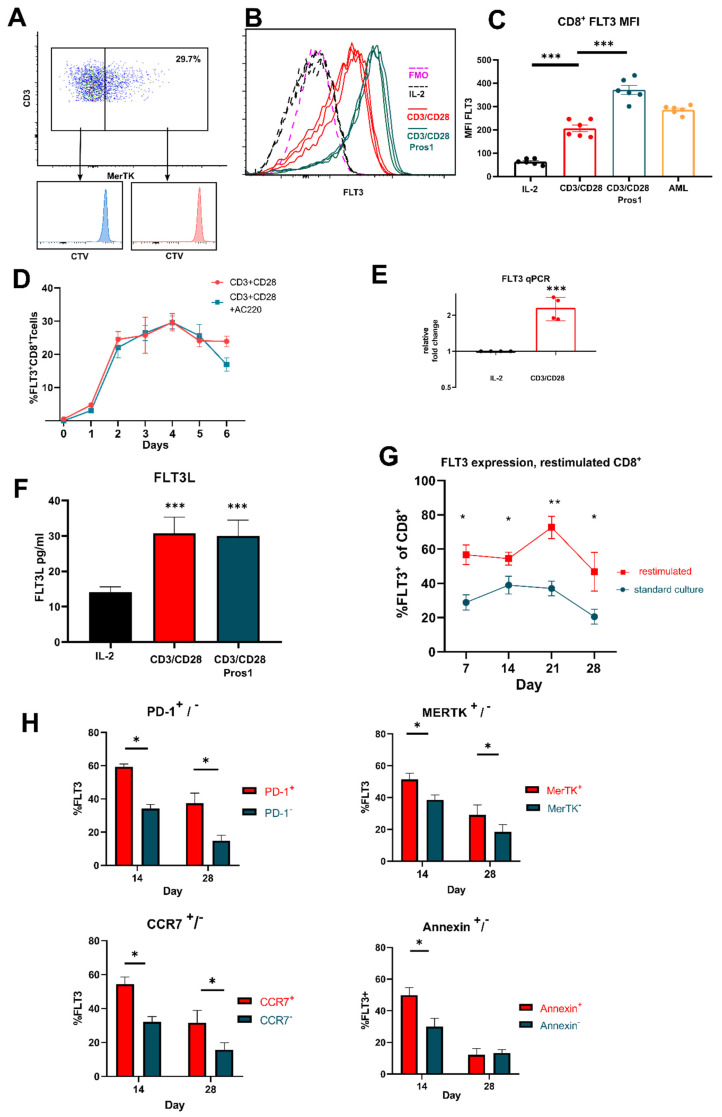
Use of dual MerTK/FLT3 inhibitor UNC2025 reveals CD8 T cell expression of FLT3 following activation. CD8^+^ T cells were activated with CD3/CD28 + IL-2 50 U/mL. (**A**) Representative dot plot revealing inhibited cell division in MerTK+ and MerTK− CD8^+^ T cells when cultured with UNC2025. (**B**) Representative histogram of 3 replicates showing MFI of FLT3 expression in 3-day activated CD8^+^ T cells compared to non-stimulated, FMO and activated CD8^+^ T cells cultured with additional 50 nmol/l Pros1. (**C**) MFI of FLT3 in 3-day activated CD8^+^ T cells, compared with addition of 50 nmol/l Pros1 and AML THP-1 cell line. (**D**) Percentage expression of FLT3+ CD8^+^ T cells following activation measured day 0 to day 6, in presence or absence of addition of 750 nM AC220. (**E**) RT qPCR evaluated expression of FLT3 from 3-day activated CD8^+^ T cells, normalized against non-stimulated CD8^+^ T cells. (**F**) Concentration of FLT3L in supernatants of non-stimulated, 3-day activated CD8^+^ T cells, and with addition of 50 nM Pros1. (**G**) FLT3 expression of activated CD8^+^ T cells during long term culture, and restimulation, measured day 7, 14, 21, and 28. (**H**) Percentage of FLT3+ cells in MerTK +/−, PD-1 +/−, CCR7 +/−, and Apoptotic +/− populations of CD8^+^ T cells following activation and longterm culture. Data are plotted as mean and +/− SEM where applicable. Statistical significance is calculated using Students *t* test or One-Way Anova, * *p* ≤ 0.05, ** *p* ≤ 0.01, *** *p* ≤ 0.001.

This complete cell cycle arrest by MERTK inhibition led us to further question the specificity of UNC2025, as only approximately 30% of CD8^+^ T cells express MERTK, [25,35]. Figure 3A demonstrates that both MERTK positive and MERTK negative cells had restricted cell division, suggesting UNC2025 potentially targeted other receptors on activated T cells. As UNC2025 is a dual MERTK/FLT3 inhibitor, we therefore investigated expression of FLT3 on activated T cells (Figure 3B,C). To our surprise, FLT3 was indeed expressed by three-day activated T cells but not by unstimulated T cells. Furthermore, with the knowledge that addition of PROS1 into the culture of activated T cells leads to increased activation and proliferation, we found that PROS1 increased FLT3 expression (Figure 3C). To investigate FLT3 expression kinetics, time-course experiments demonstrated that peak expression is between day three and day four with 30.23% of CD8^+^ T cells expressing FLT3 (Figure 3D). FLT3 mRNA expression was confirmed in activated CD8^+^ T cells by real-time qPCR (Figure 3E). Reflecting similar kinetics to MERTK expression and PROS1 production, we also showed that three-day activated CD8^+^ T cells secreted FLT3 ligand (Figure 3F). Long term culture of CD8^+^ T cells activated by CD3/CD28 revealed sustained expression of FLT3 for 28 days with a peak expression of 38.98% FLT3+ on day 14, dipping to 20.55% by day 28 (Figure 3G). Separately, T cells from these cultures were reactivated once at various timepoints. Restimulation increased FLT3 expression with a peak of expression at day 21 of 72.71% FLT3+ (Figure 3G). Next, to assess any associated activation-induced phenotype with FLT3 expression, we analyzed PD-1+, CCR7+ and MERTK+ CD8^+^ T cells on day 14 and 28 culture (Figure 3H). Greater FLT3 expression was associated with positive fractions of these cell phenotypes at all time points, with greatest differential of FLT3+ CD8^+^ T cells on day 14. This could suggest that FLT3 expression is associated with activation status and memory with increased proliferative capacity. Additionally, we investigated FLT3 expression and association with activation-induced cell death (AICD). On days 14 and 28, of long-term cultured CD8^+^ T cells were reactivated once and cultured for a further two days prior to analysis. FLT3 expression was expressed significantly higher upon restimulation in apoptotic cells (annexin high) on day 14 hinting that FLT3-signaling could be associated with activation and cell division, playing roles in the expansion phase of T cell proliferation.

### 3.3. FLT3 Inhibitor AC220 Impacts CD8^+^ T Cell Cycle Kinetics

**Figure 4:**
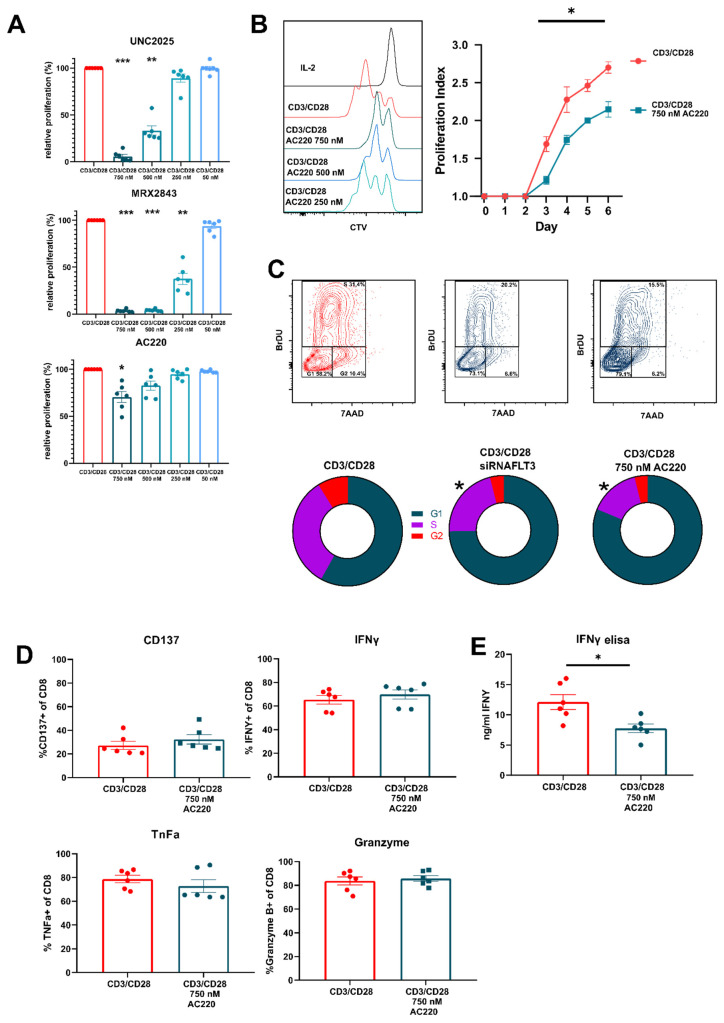
Use of FLT3 inhibitor AC220 (Quizartinib) affects rate of cell division in activated CD8 T cells. CD8^+^ T cells were activated with CD3/CD28 + IL-2 50 U/mL and cultured in presence or absence of AC220, MRX2843 or UN2025. (**A**) Relative percentage of cell division in 3-day activated CD8^+^ T cells cultured in 750 nM, 500 nM, 250 nM, and 50 nM of UNC2025, MRX2843 and AC220. (**B**) Proliferation index measured day 0 to day 6 of activated CD8^+^ T cells. (**C**) Representative contour plots and percentage proportion of cell cycle populations G1, S and G2 phase in 3-day activated CD8^+^ T cells transfected with either FLT3 siRNA or Mock siRNA or cultured with 750 nM AC220 (**D**) Percentage of CD137, IFNY, Tnfa, and Granzyme B positive CD8^+^ T cells following 3-day activation. (**E**) Concentration of IFNy from supernatants of 3 day-activated CD8^+^ T cells. Data are plotted as mean and +/− SEM where applicable. Statistical significance is calculated using Students *t* test, One-Way Anova, or Two-Way Anova * *p* ≤ 0.05, ** *p* ≤ 0.01,*** *p* ≤ 0.001.

Having concluded that FLT3 is expressed by activated T cells we went on to examine the effect inhibition of FLT3 has on activated CD8^+^ T cells. We initially compared the effect of dual MERTK/FLT3 inhibitors UNC2025 and MRX2843 against that of AC220 (Figure 4A). We found that whilst dual inhibitors had a very potent effect on cell division, AC220 had a moderate but significant effect on proliferation at 750 nM where relative proliferation was reduced to 70.58% to that of control. When analyzing proliferation index, whereby cell divisions are measured only in dividing cells, we discovered that inhibition of FTL3 had a significant impact reducing rate of cell division by approximately 30% (Figure 4B). Through cell cycle analysis, we found that AC220 inhibited cell cycling at the G1/S phase. To confirm this, we performed siRNA-mediated knockdown of FLT3 on day 3 activated CD8^+^ T cells. When compared to control, we found a similar effect in accumulation of cells in G1/S phase to that of FLT3-inhibitor AC220 (Figure 4C). Due to the stimulatory effect that MERTK ligand PROS1 has on activated T cells, we tested whether the addition of FLT3L to activated CD8^+^ T cells had a similar effect as PROS1. Total proliferation and proliferation index were not affected by concentrations of FLT3L tested (Supplementary Figure S4B). Finally, as with UNC2025, we tested whether AC220 affected cell activation and intracellular cytokine production (Figure 4D). We demonstrated that inhibition of FLT3 did not affect expression of CD137 or per cell cytokine production; however, total secreted amounts of IFN-γ were decreased (Figure 4E). This suggests that, as with UNC2025, FLT3 inhibition and its reduction on rate of cell division ultimately affects total cytokine released.

## 4. Discussion

Our results demonstrate that use of RTKIs specific for MERTK and/or FLT3 induce human CD8^+^ T cell cycle arrest. This effect is distinct to that of activation and does not promote apoptosis. This phenomenon may be due to their role as costimulatory or proinflammatory receptors, where feedback loops promote cell proliferation. As cytokine production on a cell by cell basis is not affected, this suggests that inhibition of MERTK and FTL3 is restricted to signaling pathways such as mTOR, preferentially involved in cell division [36].

Full characterization of function of FLT3 and FLT3L expression by T cells has not been done until now, despite earlier studies reporting expression [30,31]. Due to the potency of UNC2025 and its effect on both MERTK+ and MERTK− cell populations, we concluded that it was within reasonable doubt to examine FLT3 expression as a receptor on activated T cells. We were able to show that FLT3 is indeed expressed by activated T cells, in a similar pattern as MERTK. FLT3 expression was related to MERTK, PD-1 and CCR7expressing cells, suggesting that FLT3 is widely expressed amongst activated and proliferating cell subsets. Addition of FLT3L to culture did not alter cell proliferation kinetics, whereas small molecule inhibitors and siRNA knockdown did impact cell cycling negatively. Further investigation into the role of FTL3 signaling induced by FLT3L in mature T cells would provide a crucial insight into how this signaling pathway is related to T cell activation, maturation and proliferation and further inform us of how this signaling pathway can be navigated in anti-tumoral immunotherapy.

Wolleschak et al. demonstrated that 50 nM of AC220 did not have any substantial effect on T cell proliferation or function in vitro [37]. In this study, human primary T cells were cultured or activated for approximately two days prior to inhibitor addition or analysis. We report that the window of FLT3 expression only starts at day two, explaining the possible discrepancies. In addition, it is important to note our study makes use of concentrations of 500 nM or greater. These concentrations are substantially greater than that used in original studies on the efficacy and characterization of AC220 and UNC2025, where concentrations as little as 10 nM caused inhibition of phosphorylation of their targets [38,39]. Such small concentrations caused a significant effect on some in vitro tumor cell lines; however, concentrations of 500 nM and above demonstrated detrimental effects on colony forming units in cord blood and bone marrow [6,11,40,41,42]. Furthermore, many in vivo and clinical trials used much greater doses, where serum concentrations reached 3 µM per day [43,44,45,46,47]. Our results demonstrate a higher threshold of tolerance from activated CD8^+^ T cells towards MERTK and FLT3 inhibitors. This allows for further consideration for the optimal dose of inhibitors to be able to demonstrable clinical effects, whilst not impacting normal immune cell function in patients.

Our studies highlight that there is much to discover in the repertoire of receptors expressed by T cells, especially in non-resting activated states. Indeed, a recent discovery by Frumento et al. highlights that activated CD8^+^ T cells express c-KIT, an RTK of the same family as FLT3, and that its function is inherently tied to T cell priming and propensity to apoptosis [48]. With the rapid development of a new generation of RTKIs, it is important to consider the impacts these potent inhibitors may have on anti-tumor immune responses. We showed that use of UNC2025 increased PD-1-high expression on activated T cells. This increase is likely linked to the abrogation of normal T cell maturation and proliferation kinetics resulting in increased expression of PD-1 and potential T cell exhaustion. Indeed, this may be related to similar findings in in vivo studies where PD-1 blockade is required when using MERTK inhibitors, to achieve anti-tumor efficacy [32,33,34]. Moreover, we showed that UNC2025-mediated inhibition resulted in decreased mTOR phosphorylation. The mTOR pathway is tightly involved in the process of T cell growth and proliferation [49]. Interestingly, we observed a hyperactivation of ERK1/2, likely as a compensatory mechanism as cell activation and cytokine production remained unaffected. Paradoxal hyperactivation of ERK upon small molecule inhibition in T cells has been described before [50]. Finally, the observed cell cycle arrest of T cells when using UNC2025 or AC220 may also impact inflammatory cytokine profiles at the tumor microenvironment. Our work highlights that despite no restriction in cell activation or cytokine production, we still observed significant reduction in IFN-γ in supernatants. This suggests that fewer cell numbers will ultimately affect localized concentration of anti-tumoral cytokines, even though intracellular cytokine expression assays do not show any changes.

The duality of anti-tumor effects and anti-immune cell activity that some RTKIs present should be taken into consideration when designing appropriate studies to examine off-target effects, especially into cell cycle kinetics and proliferation. Only limited conclusions can be made when examining effective concentrations against cancer cell lines in vitro, and concentrations often do not reflect serum concentrations seen in vivo or in clinical studies. We suggest that RTKIs should be tested in human primary T cells in a range of concentrations, given the importance of this cell subset to the outcome of anti-tumor immune responses. Alterations of tumor-induced proliferation of CD8^+^ T cells following antigen recognition and TCR-mediated activation will have detrimental effects on the efficacy of cancer treatments.

## Data Availability

All generated data supporting reported results are included in this article.

## Supplementary Materials

The following are available online at https://www.mdpi.com/article/10.3390/vaccines9111294/s1, Table S1: Primer and siRNA sequences, Figure S1: Gating strategies for Figure 1, Figure S2: Gating strategy for phosphorylation analysis. Figure S3: Gating strategies for Figure 3. Figure S4: Gating strategies for Figure 4.
