# Supplementary material for: Small Molecule Inhibitors of MERTK and FLT3 Induce Cell Cycle Arrest in Human CD8+ T Cells
Source: Vaccines (Basel). 2021 Nov 8;9(11):1294. doi: 10.3390/vaccines9111294 (PMC8617686; doi:10.3390/vaccines9111294)
Supplement: Supplementary file 1 [file vaccines-09-01294-s001.zip › vaccines-1419837-supplementary.pdf]

Supplementary Materials

Table S1: Primer and siRNA sequences

FLT3 forward	TCACAGGGGGAAAGCTGTAA
FLT3 reverse	GCACCTTATGTCCGTCCCAA
siRNA 1 forward	GGCUGUUCACAAUAGAUCUtt
siRNA 2 reverse	AGAUCUAUUGUGAACAGCCtg
siRNA 2 forward	GGACUAUUACAAAUCAAGAtt
siRNA 2 reverse	UCUUGAUUUGUAAUAGUCCca
siRNA 3 forward	: CAACUAUCUAAGAAGUAAAtt
siRNA 3 reverse	UUUACUUCUUAGAUAGUUGag

Supplementary Figure 1

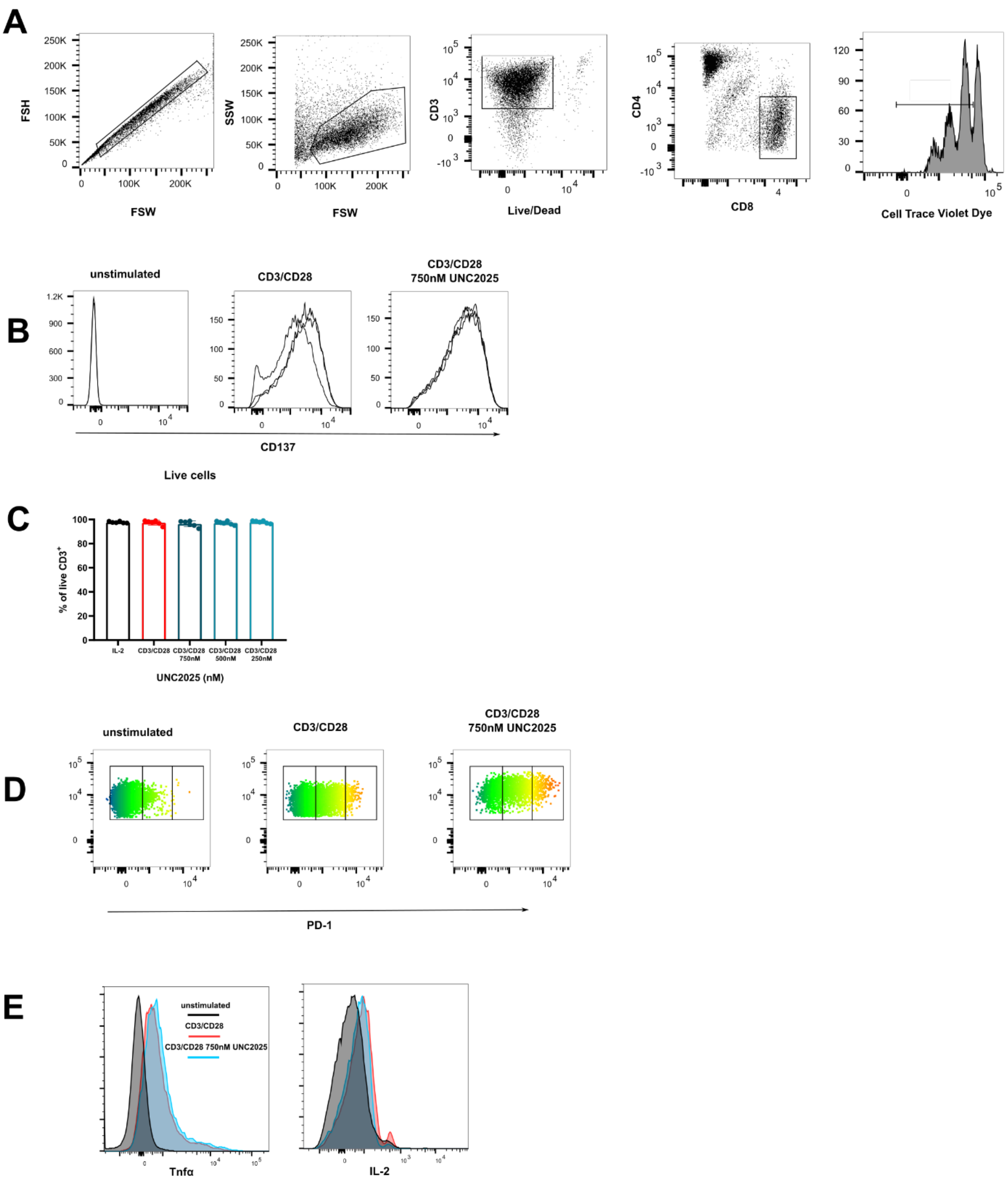

Supplementary Figure S1: (a) Flow cytometry gating method for analysis of CD8^{+} T cells. (b) Representative histogram plots CD137 expression (n=3). (c) Percentage of live T cells after 3 day culture in the presence of UNC2025(n=6). (d) Representative gating scheme for analysis of PD-1 neg/intermediate/high expression in CD8 T cells. (e) Representative histogram plots of Tnfa and IL-2 intracellular expression of CD8 T cells in culture with UNC2025.

Supplementary Figure 2

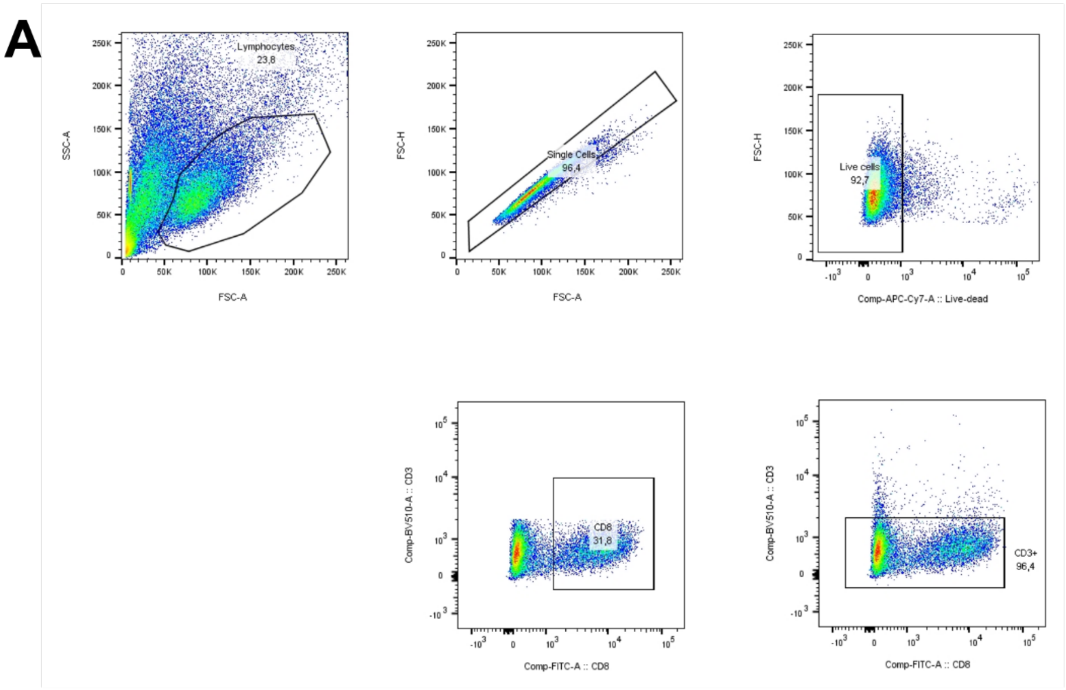

Supplementary Figure S2: (a) Representaive flow cytometry gating method for phospho-flow analysis.

Supplementary Figure 3

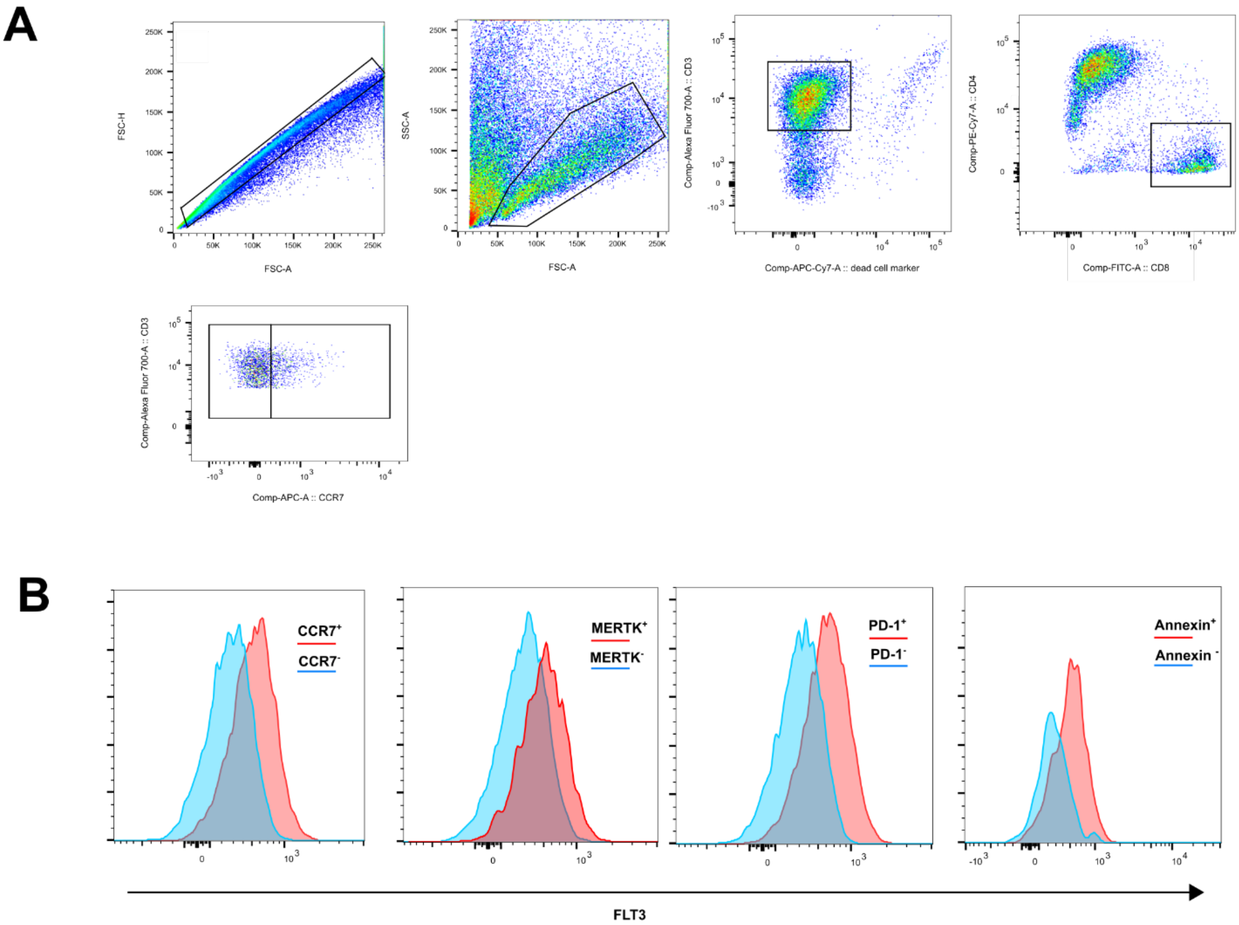

Supplementary Figure S3: (a) Representative flow cytometry gating method for gating of MERTK^{+} CD8^{+} T cells. (b) Representative histogram plots of FLT3 expression in positive and negative fractions of CCR7, MERTK, PD-1 and Annexin high T cells following CD3/CD28 activation and 14 days of culture.

Supplementary Figure 4

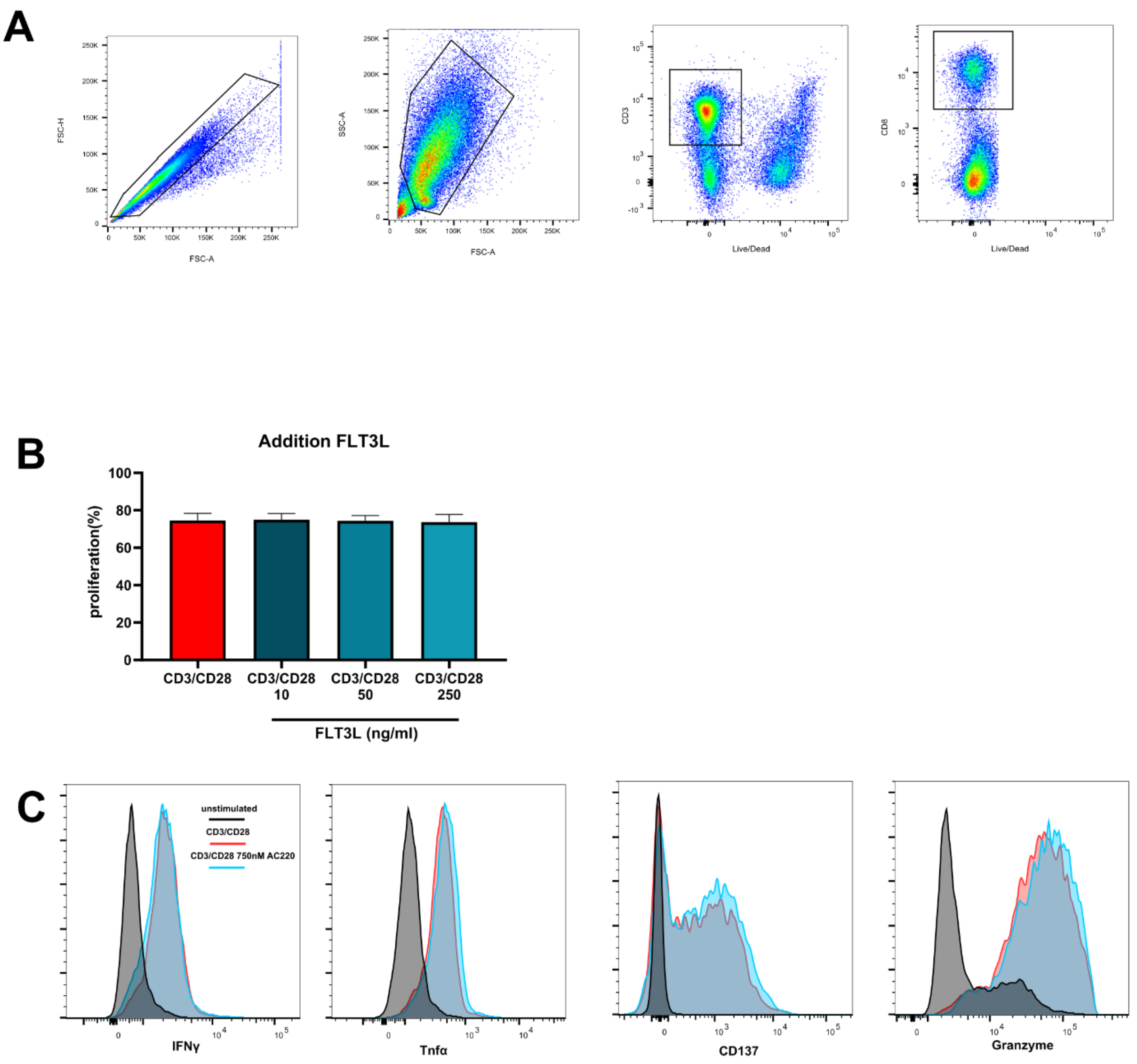

Supplementary Figure S4: (a) Representative flowcytometry gating method of CD8^{+} T cells for cell cycle kinetics using AC220 and FLT3siRNA. (b) Percentage of proliferating CD8^{+} T cells following activation and addition of FLT3L (n=6). (c) Representative histogram plots of intracellular cytokine expression of IFNγ, Tnfα and granzyme, and surface expression of CD137 follwing CD3/CD28 activation and use of AC220 in culture.
